# Chemical Contamination in Bread from Food Processing and Its Environmental Origin

**DOI:** 10.3390/molecules27175406

**Published:** 2022-08-24

**Authors:** Agnieszka Maher, Adriana Nowak

**Affiliations:** Department of Environmental Biotechnology, Faculty of Biotechnology and Food Sciences, Lodz University of Technology, Wolczanska 171/173, 90-530 Lodz, Poland

**Keywords:** Maillard reaction, bread contaminants, food processing, mycotoxins, toxic metals, pesticides

## Abstract

Acrylamide (AA), furan and furan derivatives, polycyclic aromatic amines (PAHs), monochloropropanediols (MCPDs), glycidol, and their esters are carcinogens that are being formed in starchy and high-protein foodstuffs, including bread, through baking, roasting, steaming, and frying due to the Maillard reaction. The Maillard reaction mechanism has also been described as the source of food processing contaminants. The above-mentioned carcinogens, especially AA and furan compounds, are crucial substances responsible for the aroma of bread. The other groups of bread contaminants are mycotoxins (MTs), toxic metals (TMs), and pesticides. All these contaminants can be differentiated depending on many factors such as source, the concentration of toxicant in the different wheat types, formation mechanism, metabolism in the human body, and hazardous exposure effects to humans. The following paper characterizes the most often occurring contaminants in the bread from each group. The human exposure to bread contaminants and their safe ranges, along with the International Agency for Research on Cancer (IARC) classification (if available), also have been analyzed.

## 1. Introduction

It is known how important access to water and food is; without them, humankind is not able to live or survive. Thanks to food, all the nutritious ingredients for the human organism to be strong and healthy are delivered. Human bodies use all the compounds present in food as energetic, regulatory, and building fuel. Wheat has been a base of the human diet for centuries, as reviewed previously [1]. Worldwide wheat production was estimated at approximately 768 million tons in 2021 [2], while among the biggest wheat distributors forecasted in 2021 were Asia at 278.9 million tons, followed by Europe at 268 million tons [2]. The bread’s main ingredients are flour, water, yeast, and leavening agents, distinguished in different recipes with characteristic properties [3]. Gluten and its proteins are the key factors directly influencing wheat quality during baking [4].

**Figure 1:**
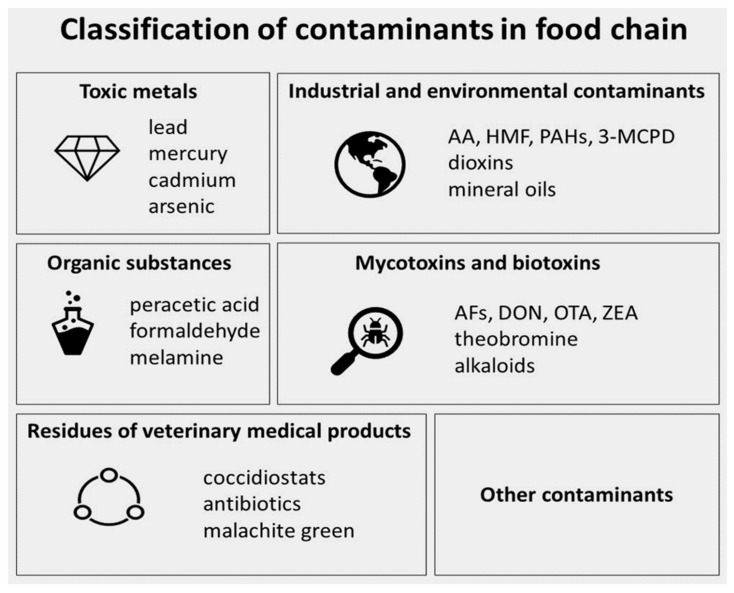
Classification of contaminants in the food chain on the basis of the European Food Safety Authority [6].

Unfortunately, foodstuffs consumed by people are also more and more often a source of food contaminants. According to Codex Alimentarius, a contaminant is defined as “any substance not intentionally added to food or feed for food-producing animals, which is present in such food or feed as a result of the production (including operations carried out in crop husbandry, animal husbandry, and veterinary medicine), manufacture, processing, preparation, treatment, packing, packaging, transport or holding of such food or feed, or as a result of environmental contamination. The term does not include insect fragments, rodent hairs, and other extraneous matter” [5]. It is reasonable to categorize food toxicants according to the European Food Safety Authority (EFSA) (Figure 1) [6]. These food chain contaminants can occur in food due to food production, distribution, packaging, and consumption, but also can be present naturally in the environment. Another aspect is food additives, which are being added to modern food to improve organoleptic qualities but also to prevent spoilage by prolonging foodstuffs’ shelf life (e.g., preservatives, decorative food additives, fortifying agents), as mentioned in the case study of Bimpizas-Pini et al. [7]. According to Codex Alimentarius, a food additive is defined as “any substance not normally consumed as a food by itself and not normally used as a typical ingredient of the food, whether or not it has nutritive value, the intentional addition of which to food for a technological (including organoleptic) purpose in the manufacture, processing, preparation, treatment, packing, packaging, transport or holding of such food results, or maybe reasonably expected to result, (directly or indirectly) in it or its by-products becoming a component of or otherwise affecting the characteristics of such foods” [8]. Additionally, there are also other harmful groups of contaminants present in food products. Food contaminants can also originate from the environment (environmental contaminants) or natural habitat (natural contaminants), or can be the result of human influence (anthropogenic source, xenobiotics).

The present review raises various groups of food contaminants occurring mainly in grain-derived products (bakery goods mostly focusing on bread) originating from (1) industry during food processing—baking and cooking; (2) environmental natural (non-anthropogenic) sources such as MTs, and (3) environmental pollution as the cause of anthropogenic activity: TMs and pesticides. Moreover, human exposure to contaminants, together with the margin of exposures (MOEs), benchmark dose lower confidence limits (BMDLs), tolerable daily intakes (TDIs), tolerable weekly intakes (TWIs), and Maximum Residue Limits (MRLs) (according to available data) were also discussed. The review has been conducted mainly with a focus on publications and research from the last 5 years. So far, no review article captured such an extensive and differentiative range of contaminants in bread, counting sources and safe ranges proposed by international organizations along with IARC classification.

## 2. Methodology

The review was prepared with the application of databases such as: PubMed, Elsevier, Wiley Online Library, Taylor Francis, Springer Link, Google Scholar, and the websites of various national and international public health organizations such as: EFSA, European Commission (EC), European Union (EU), Food and Agriculture Organization (FAO), World Health Organization (WHO), Joint FAO/WHO Expert Committee on Food Additives (JECFA), IARC, United States Environmental Protection Agency (US EPA), United States Department of Agriculture (USDA), and Agency for Toxic Substances and Disease Registry (ATSDR), up to 28 March 2022. The searched keywords were: bread, food additives, food contaminants, bread toxins, bread carcinogens, bread processing contaminants, acrylamide/furan/5-hydroksymetylo-2-furfural (HMF)/PAHs/MCPDs/monochloropropanediols esters (MCPDEs)/glycidol/glycidyl esters (GEs)/mycotoxins/metals/toxic metals/pesticides: in bread; in grains; in wheat; distribution; risk exposure. Only English-language articles were included. The full texts were accessed via the Lodz University of Technology Library.

## 3. Maillard Reaction as a Source of Bread Processing Contaminants

**Figure 2:**
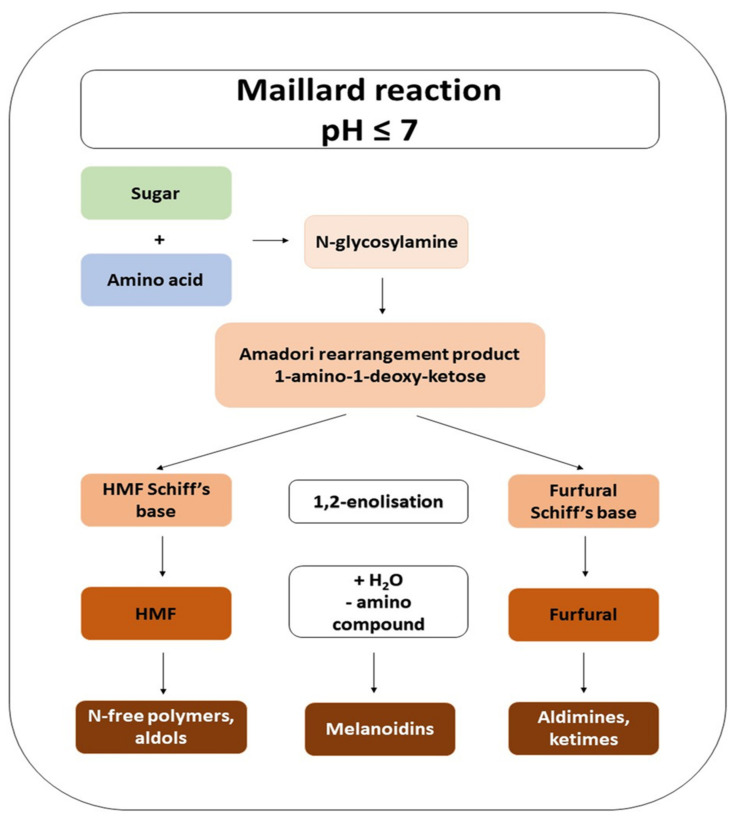
Maillard reaction mechanism modified on the basis of Singla et al. [17].

More than 500 compounds have been detected in the aroma fraction of bread, including acids, alcohols, aldehydes, esters, furans, hydrocarbons, ketones, lactones, pyrazines, and pyrroles originating from the Maillard reaction and lipid oxidation, with a third pathway resulting from yeast fermentation [9]. Louis-Camille Maillard described the glycation process of carbohydrates for the first time in 1912 (Figure 2) [10]. Since then, studies have provided significant knowledge about the primary stages of the Maillard pathway, but the mechanism of intermediary compound formation still needs a better understanding. With the advancement of reactions, a great number of carbonic compounds and polycarbons are being created. Aldehydes and ketones are formed due to the reaction between α-dicarbonyl and amino acids, known as Schiff’s base (Strecker degradation). At this stage, all reaction products are colorless. At the definitive step of the Maillard reaction, the dehydration of sugars takes place, and furan derivate is gained. This derivative reacts with other components to polymerize and leads to the generation of dark-brown, insoluble, and nitrogenous colloids called melanoidins, as reviewed by Favreau-Farhadi et al. [11]. The Maillard reaction rate depends on the structure and number of amino acids, reducing sugars, temperature, water activity, moisture content, pH, and the presence or absence of a catalyzer and inhibitor. Moreover, it has already been reviewed that the Maillard reaction rate rises when moisture and temperature are leveled up [12,13]. Previously, bread was a key product for Maillard reactions due to its medium moisture content and applied high baking temperatures. In the review of Pozo-Bayón et al. [14], it was assumed that only a small part of Maillard reaction compounds plays a significant role in the final bread aroma. Thermal processing induces a significant reaction in flavor and taste, and several unfavorable Maillard-reaction-derived chemical hazards—such as AA and heterocyclic aromatic compounds—are simultaneously formed [15]. One of the better-known compounds of these compounds is AA, a neurotoxin and potential carcinogen in humans. Moreover, volatile furan and furanic compounds have received considerable attention due to their hepatotoxic activity [16].

### 3.1. Acrylamide

**Figure 3:**
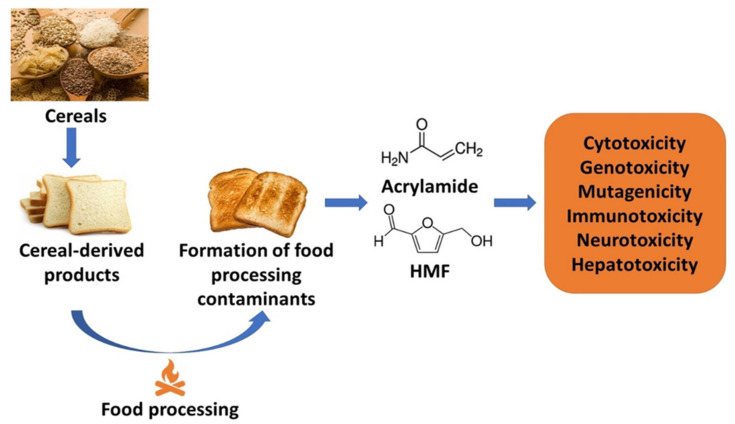
Humans’ exposure to food processing contaminants induces cytotoxicity, genotoxicity, mutagenicity, immunotoxicity, neurotoxicity, and hepatoxicity.

AA is a substance formed during the cooking process at temperatures above 120 °C [18] in foodstuffs with simultaneously high levels of asparagine (Asn) and reduced sugars, and it has not been detected in boiled food [18,19]. Nonetheless, a great gain in its production occurs in food cooked at a temperature above 170 °C [20,21]. Its mechanism of formation mainly involves the Maillard reaction. The zenithal levels of AA are common in various baked foods, together with potato-based food, savory snacks, starchy products, and roasted coffee beans, but also in cigarette smoke and cosmetics [22,23,24,25]. AA formation in baked bread can be determined by many factors, such as moisture level, pH, temperature, and time. High temperature and low moisture content during the Maillard reaction promote chemical reactions between food components which change the properties of the final product [26]. A lot of desired compounds develop organoleptic characteristics, e.g., the flavor and color of the baked product. However, due to the high temperature required for this process, the formation of AA in these products is an inevitable negative effect and real-time human health concern (Figure 3) [27,28]. It has been proven that AA influences neurotoxicity by the deletion of the Nrf2 gene [29]. AA neurotoxicity was also observed by diminished ATPase activity, enhanced activity of acetylcholinesterase, and dopamine depletion [30]. AA has stimulated elevated levels of pro-inflammatory cytokines such as tumor necrosis factor-alpha (TNF-α) and interleukin-1β (IL-1β) [31]. AA may act as a colon co-carcinogen in association with an azoxymethane carcinogen [32]. The glycidamide (GA) compound is the main AA metabolite. It has been found that GA is more reactive than AA and, at low doses, is more potent to form tumors than AA [33]. AA has been reported to be a human neurotoxin, a rodent carcinogen (group 2A), and a probable carcinogen to humans [34].

The EFSA Panel on Contaminants [35] concluded that there was no sufficient evidence to follow any hypothesis which would be related to AA exposure directly resulting in an increased risk for any major cancer type. However, after the risk evaluation of AA present in thermally heated food, the Contaminant Panel, influenced by the recent scientific data and performed studies, provided a dietary exposure assessment on AA, based on the MOE approach on neurotoxic and carcinogenic effects. The estimation of MOEs was made upon the benchmark dose lower confidence limit for a 10% increase in the number of tumor-bearing animals in comparison to control animals (BMDL10), and was calculated by dividing the BMDL10 values by the mean and high-level estimates of dietary exposure to AA. The EFSA set up the lowest value among BMDL10 as the accepted value. This risk evaluation showed that AA is present on a large scale in heat-processed foods, and this exposure to AA through diet is responsible for an increased risk of developing cancer in consumers of all age groups [35]. During the meeting, four possible critical limits for AA toxicity were identified: (I) neurotoxicity, (II) effects on male reproduction, (III) developmental toxicity, and (IV) carcinogenicity. The recent data from animal studies could be used to measure the reference AA dosage. The following BMDL10 reference points were proposed as follows: 430 μg/kg body weight (BW)/day for peripheral neuropathy in rats, and 170 μg/kg BW/day for neoplastic effects [35]. Based on these reference points, the EC adopted Regulation 2017/2158, dedicated to mitigation measures and benchmark levels for a reduction in the AA presence of certain foodstuffs [36]. According to this regulation, the levels of AA in wheat-based soft bread and crispbread should not exceed 50 and 350 µg/kg, respectively. The maximum limit for biscuits and ice cream wafers is 300 µg/kg, whereas the threshold for other products (e.g., sweets) is 350 µg/kg [36]. A comprehensive review of up-to-date obtained experience has been recently summarized by FoodDrinkEurope in the Acrylamide “Toolbox” [37]. Consumer exposure to AA depends on the levels of AA formed in foods and the presence of specific food products in the consumer’s diet [35]. The recent studies on food processing contaminants that contribute to various bread-related products are summarized in Table 1. Additionally, in Table 2 are data concerning dietary exposure to food processing contaminants in bread products in various countries.

According to Table 1, in one of six studies, the detected AA level was 87 μg/kg, and that concentration of AA in wheat-based bread is above the limit allowed by EFSA [22]. Comparing BMDL10 reference points for AA and exposure to AA gathered in Table 2, it is discovered that AA concentrations were in the safe ranges for the peripheral neuropathy and neoplastic effects. AA levels, measured in bread-related products and the sweets/biscuits group, were in the range of 31–454 and 204–400 µg/kg, respectively. The control wheat bread crust sample contained 65 µg/kg of AA [55]. Interesting but inconsistent results were obtained by Surdyk et al. [56] and Wang et al. [57]. Namely, Surdyk et al. [56] measured the AA level in yeast-leavened wheat bread baked at 270 °C for 15 min, and the concentrations of AA in the crust and crumbs were 80 and 24 µg/kg, respectively, while Wang et al. [57] obtained significantly elevated levels of AA in wheat bread baked at 220 °C for 25 min, 570 and 270 µg/kg, for the crust and crumb, correspondingly. Nevertheless, Surdyk et al. [56] suggested that AA, which is detected in crumbs, originated from the parts of the crust as a result of an incomplete separation of the crust. More importantly, due to the low thermal conductivity of the dough, the inner temperature of the crumb does not reach 100 °C.

### 3.2. Furan and Furan Derivatives

In addition to AA, during the thermal processing of foodstuffs, a furan compound is formed from sugars under acidic conditions [58]. An alternative route of HMF formation in dry conditions, from fructose and sucrose, was observed using a highly reactive fructofuranosyl cation that can be transformed directly into HMF [59]. Nevertheless, the HMF carbonyl group and Asn presence can play an important role in AA formation during the Maillard reaction, while there are low moisture conditions and elevated temperatures [60]. According to the available literature, ascorbic acid, polyunsaturated fatty acids (PUFAs), carotenoids, sugars, and amino acids are the precursors for furan and its derivatives’ formation. Precisely, furan and 2-methylfuran (2MF) are produced from carbohydrate precursors in two distinct pathways. Furan forms directly from carbohydrate degradation, while 2MF mostly forms from the condensation of carbohydrate moieties generated during the Maillard reaction [61,62]. The furfural is a compound with a characteristic almond odor and has been primarily established by the 1,2-enolisation pathway via 3-deoxyosone, but may also be formed during the fermentation process [63]. Moreover, the crucial role in the development of furan and furan derivatives in foodstuffs such as coffee, canned meat, baked bread, and hazelnuts is the thermal-oxidative degradation of PUFAs and ascorbic acid [64]. In animal studies, HMF is potent to undergo biotransformation into the genotoxic and mutagenic metabolite, sulphoxymethylfurfural [65,66], while furfural may lead to hepatotoxicity [67]. It was already found before that furan is highly potent with regard to exerting carcinogenic and mutagenic effects in rats and mice, probably due to cis-butene-1,4-dial reactive metabolites, which originate from furan oxidation by cytochrome P450 and bind to proteins and nucleosides [68,69,70]. In 1995 the IARC classified furan, together with furfural in the Group 2B, as “possibly carcinogenic to humans” [71]. Additionally, 2MF has been announced to produce highly reactive intermediates, likewise to furan, leading to similar toxicity in the liver of rats [72]. Recently, there have been a great number of studies conducted that focused on hydroxymethylfurfural (5-HMF), which consists of a furan ring with an aldehyde and an alcohol group and is formed as an intermediate product during non-enzymatic browning reactions [73,74,75]. There are available studies that observed that 5-HMF is potent to cause eye, respiratory tract, and skin irritation, and can be carcinogenic, hepatotoxic, nephrotoxic, or lead to neoplastic transformation [66,75,76].

The EFSA Panel on Contaminants [77] has chosen the BMDL10 of 64 μg/kg BW/day and 1310 μg/kg BW/day as reference points for the risk characterization of non-neoplastic and neoplastic effects induced by furan. Because of the lack of a direct genotoxic mechanism in the carcinogenic mode of action of furan, the Contamination Panel decided that it was not suitable to announce a TDI, and the MOE approach has been introduced instead. The MOE value for the neoplastic effects was set above 10,000. The calculated MOEs showed that no group is at risk of developing non-neoplastic effects, and the exposure levels for all the population groups were above 100. As a consequence of not having enough information, the health risks associated with the dietary intake of 2MF and 3-methylfuran (3MF) could not be characterized [77].

There are no data concerning permissible limits of furan and furan derivatives in wheat-based products. However, comparing data collected in Table 2 with the regulations of EFSA, it has been shown that levels of HMF and 5-HMF were 87,000 and 12,000 μg/kg BW/day, respectively, being much above the accepted concentrations for BMDL10s for neoplastic effects [22,54]. In the Mildner-Szkudlarz et al. [55] study, furan derivatives such as furanmethanol, 2-acetylfuran, and 5-methylfurfural have been found in the wheat loaf system. The decomposition of glycosylamine at the beginning of the chemical reaction results in Maillard-type furanic compounds forming. Additionally, 5-methylfurfural was found in the blank crust at a concentration of 38 µg/kg, while furfural was present in the blank bread crust at a level of 565 µg/kg [9].

### 3.3. Polycyclic Aromatic Amines

Other compounds of food processing contaminants are PAHs. The PAHs have been differentiated into two groups: (1) from one to four benzene rings are known as light polycyclic aromatic hydrocarbons (L-PAHs), and (2) those containing more than four benzene rings are known as heavy polycyclic aromatic hydrocarbons (H-PAHs). H-PAHs are more stable and toxic than L-PAHs [78]. The presence of PAHs in food can be from natural (as in environmental) and synthetic sources (food processing). Even though PAHs are known to be typical heat-induced food toxicants mainly formed in high-protein foodstuffs, e.g., meat or fish, their presence has also finally been reported in some baked bread systems, and they were extensively reviewed [79]. The cooking method is crucial in the formation of PAHs in foods. As well, thermally induced processes can lead to PAHs contamination even higher than traffic [80]. The PAHs content may be affected by the unsuitable drying method of cereals, seeds, and edible oils [81]. Depending on grilling conditions, grilling direction, grilling distance, and the use of different fuels can lead to different compounds of PAHs [82]. The highest benzo[a]pyrene (BaP) level in barbecued food is present when charcoal with wood chips is used as fuel [83]. Commonly, the highest concentration of PAHs was detected in charcoal-grilled, flame-gas-grilled, and oven-grilled dishes [84]. According to the EFSA [85], the highest contributor to dietary exposure to BaP was cereals and cereal-derived products—24%. Additionally, it has been demonstrated that PAHs influence reactive oxygen species (ROS) generation, leading to inflammation and apoptosis [86,87], and induce genotoxic, mutagenic effects [88,89]. Not without reason, a long list of specific PAHs were classified in: the Group 1 as “carcinogenic to humans” (BaP); the Group 2A as “probably carcinogenic to humans“ (cyclopenta[c,d]pyrene, dibenzo[a,l]pyrene); and the Group 2B as “possibly carcinogenic to humans” (5-methylchrysene; benzo[b]fluoranthene, benzo[c]phenanthrene, benzo[k]fluoranthene, benzo[j]fluoranthene, chrysene; dibenzo[a,i]pyrene, dibenzo[a,h]pyrene, indeno [1,2,3-c,d]pyrene, naphthalene) [90,91,92]. According to Commission Recommendation 2005/108/EC [93], it has begun to be necessary to measure the presence of BaP and other listed genotoxic PAHs compounds in food products. The BMDL10 of 0.07 mg/kg BW/day was chosen for BaP, and the BMDL10 of 0.34 mg/kg BW/day for chrysene, benz[a]anthracene, and benzo[b]fluoranthene. The MOE values for high consumers ranged from 9600 to 10,800 [85].

Nevertheless, the number of consumed bread is relatively increasing, and the maximum levels of PAHs have not been established. In addition, studies concerning the problem of bread toxicants are mainly focused on AA and furanic compounds. In the study of Chawda et al. [94], it has been observed that a total of 16 PAHs were present in tandoori and Tawa bread, which ranged between 113–211 µg/kg and 60–77 µg/kg, respectively. Al-Rashdan et al. [78] detected BaP in 8 of the 18 samples in the range of 3–17 μg/kg in white wheat bread, while the total PAHs levels varied from 1 to 44 μg/kg and from 3 to 279 μg/kg for H-PAH and L-PAH, respectively. Orecchio et al. [95] have estimated the daily intake of total PAHs, which was based only on a daily consumption of 300 g of bread per person, where PAHs consumption ranged from 2 to 69 µg/day for the bread baked with wood as fuel. Even though the PAHs levels have been significantly high in the mentioned results, there are too few studies concerning its presence in bread systems.

### 3.4. Monochloropropanediols, Monochloropropanediols Esters, and Glycidyl Esters

Fatty acid esters of MCPDs and glycidols are emerging process contaminants that are mostly present in oil-containing products, but also were observed in starchy food matrixes [96]. Depending on chlorine localization, MCPDEs can be differentiated into two groups: 3-monochloro-1,2-propanediol esters (3-MCPDEs) and 2-monochloro-1,3-propanediols esters (2-MCPDEs). The 3-MCPDEs were first noted in foodstuffs by Svejkovská et al. [97], and two years later, have gained great attention after their detection in refined seed olive oil at the skyrocketing level of o 2462 µg/kg [98]. Both MCPDEs are formed in the reaction between lipids (mono-, diacyl-, and triacylglycerol, and glycerophospholipids) and chlorine donors such as sodium chloride that may be naturally present or intentionally added at high temperatures, mainly during the deodorization process [99]. The GEs are formed from mono- and diacylglycerol after the elimination of water or fatty acids at temperatures higher than 200 °C. Another possible path for GE formation is from MCPD monoesters after the elimination of hydrochloric acid [100,101]. The 2-MCPDEs, 3-MCPDEs, and GEs undergo hydrolysis in the gastrointestinal tract into their corresponding free forms (2-MCPD, 3-MCPD, and glycidol, respectively). Due to their potential toxicological effect on humans, the IARC has classified glycidol as a probable carcinogen (Group 2A) with a genotoxic and carcinogenic effect, and 3-MCPD as possibly carcinogenic (Group 2B) with non-genotoxic effects and reduction abilities on male fertility based on animal studies [102,103]. Since then, a great number of studies have been focused on the 3-MCPDE formation mechanism.

The EFSA Panel on Contaminants [104] established a TDI of 0.8 μg/kg BW/day for 3-MCPD and 3-MCPDE. No TDI could be established for 2-MCPD, 2-MCPDE, and glycidol due to the lack of toxicological information [104]. However, the MOE method was chosen to evaluate the risk to glycidol. It was presumed that hydrolysis of the esters into free glycidol occurs upon ingestion. Instead of BMDL, a 25% increase in the incidence of a specific tumor above background incidence in the lifespan of the species procedure was used. The reference point used for glycidol was 10,200 μg/kg BW/day. Nevertheless, the panel considered that an MOE of 25,000 or higher was enough to conclude that there was no health concern [104].

In accordance with Table 1, the 3-MCPD level in bread was equal to 120 μg/kg [44]. The concentration of 120 μg/kg divided by a 70 kg person gives a final concentration of 1.7 μg per person. The obtained value is above the TDI proposed by EFSA, while glycidol content in bread was in the safe range [44]. Higher concentrations of 3-MCPDEs were observed in the crust and toasted white bread at levels of 547 and 160 μg/kg, respectively. Mentioned values were significantly higher (82 and 24 times) than the ones evaluated in nontreatment white bread (7 μg/kg). These findings have led to the conclusion that thermal treatment of bread is associated with a greater amount of 3-MCPDE formation in bread systems [105].

## 4. Environment as a Source of Bread Chemical Contaminants

### 4.1. Mycotoxins

MTs are also a crucial group of food contaminants that are present in the grains and can influence the toxicity profile of bread. MTs as secondary metabolites are produced by various filamentous fungi of the genera Alternaria, Aspergillus, Claviceps, Fusarium, or Penicillium, which are harmfully potent to animals and humans. MTs can be formed under different climatic conditions in the agroecosystem. They can be created directly on the growing crops or on remaining plant residues in the field and accumulated during harvesting and storing of the grain, as was reviewed previously [106]. In the book *Nanomycotoxicology*, the definition of MT was described, and it originated from the Greek terms “mykes” and “toxicum”, meaning fungus or mold and poison, respectively [107]. The term MT was introduced for the first time in 1962 in England, due to turkey chicks’ feed contamination with deoxynivalenol (DON) [108]. Studies show that approximately 25% of all grain products are contaminated with secondary metabolites of filamentous fungi [109]. Streit et al. [110] have found that MT contamination in feed can be high as 72%, while Kovalsky et al. [111] and Eskola et al. [112] shared the opinion that MT concentration can be estimated at 79% or more than that. Moreover, MTs can also be found in other types of food such as coffee, fruits, nuts, and spices [113]. MTs consist of a variety of chemical structures with different biological properties. The classification of MTs can be made upon their chemical structure or their origin (fungal genera), but it is vital to remember that one MT can be synthesized by several fungal species, as was reviewed by Degen [114]. More than 300 MTs have been described in the literature [115]. The most important MTs present in food products and animal feed are as follows: aflatoxins (AFs) including aflatoxin B1 (AFB1), aflatoxin B2 (AFB2), aflatoxin G1 (AFG1), aflatoxin G2 (AFG2), produced by Aspergillus species; citrinin (CIT); fumonisins (FUMs), including fumonisin B1 (FB1), fumonisin B2 (FB2), fumonisin B3 (FB3); ochratoxin A (OTA), produced by both Aspergillus and Penicillium species; trichothecenes (TCs), including DON, T-2 toxin (T-2), HT-2 toxin (HT-2), nivalenol (NIV); and zearalenone (ZEA). Most affected by the mentioned MTs are cereals such as corn, wheat, barley, oats, as well as rice [115]. There are also other groups of MTs found in grains and grain-derived products such as beauvericin (BEA), enniatins (ENs), fusaproliferin (FUS), and moniliformin (MON) that are mainly produced by Fusarium species [113]. The performed research on the MT presence in grains and grain-derivative products has shown that various MTs are present at different levels. The emerging group of MTs is called “masked MTs”. Masked MTs are MTs that interact with other compounds such as amino acids, carbohydrates, and sulfate groups in grains. Meta-analysis of Sarmast et al. [116] has shown that their identification in cereals is extremely challenging. The scope of up-to-date research on the MT presence in different wheat-based products is summarized in Table 3. Additionally, Table 4 shows data concerning risk assessments of MT distribution in grain products depending on the country.

One of the most critical fungal diseases that can negatively impact crop production worldwide is Fusarium head blight (FHB). This fungal disease is spread usually in a specific area, where the climate during the flowering stage of cereal crops is warm and wet. In the reviews of Dahl and Wilson [125] and Wilson et al. [126], it was assumed that the high production of MT levels in bread is associated with lowering the quantity and quality of bread (grain yield reductions). FHB may produce DON, which adversely influences human and domesticated animals’ health. TCs, as the most crucial MTs produced by Fusarium spp., have been associated with feed refusal, vomiting, and suppressed immune functions in humans and animals [4]. The most important fungal pathogens that are related to FHB are as follows: the Fusarium graminearum complex (FGC), and related species such as Fusarium avenaceum, Fusarium culmorum, and Fusarium poae [127]. This pathogen can produce a wide range of MTs, mainly DON and its acetylated derivatives (3-acetyldeoxynivalenol-3-ADON and 15-acetyldeoxynivalenol-15-ADON).

**Figure 4:**
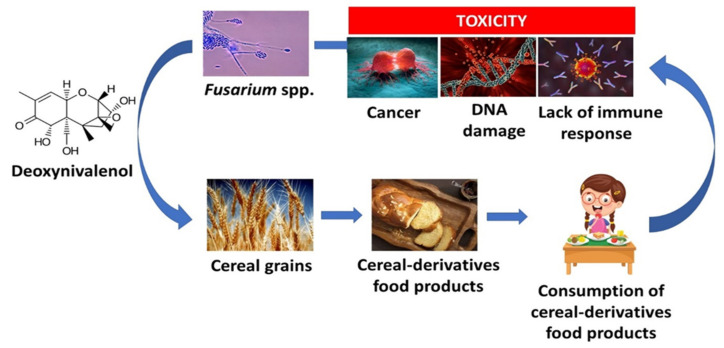
Humans’ exposure to mycotoxins through cereal-derived products can lead to cancer, DNA damage, and a lack of immune response.

MTs are unsafely and harmfully potent to both human and animal health and well-being, even after thermally processing cereals products (Figure 4). Most of the MTs are stable at high temperatures during food processing such as during baking, canning, cooking, frying, roasting, alkaline cooking, and extrusion [128]. However, MTs’ mechanism of action has not been discovered yet. Scientists proposed some hypotheses about a particular correlation between the production of secondary metabolites of filamentous fungi and responses to oxidative stress. While infection initiates, the ROS can influence fungoes pathways that are responsible for MT formation [129]. It has been proved that MTs cause acute toxicity (for example, an estrogenic effect) and long-term effects, namely carcinogenesis, mutagenesis, teratogenesis, or immunotoxicity in mammals. Humans are mainly subjected to secondary metabolites of filamentous fungi by the consumption of cereals and cereal-derived products [113]. The following AFs (AFB1, AFB2, AFG1, and AFG2) were already reported to be hepatotoxic and genotoxic, and they are classified as human carcinogens by the IARC [130]. AFs are also known as immunotoxic agents, as fetuses exposed to them in utero can cause negative effects on the growth and development of children [131]. According to the Commission Regulation 1881/2006, the maximum level for AFs in cereals was set to 2 µg/kg for AFB1 and 4 µg/kg for the sum of AFB1, AFB2, AFG1, and AFG2 [132]. FB1 has been classified as a probable human carcinogen [90], and its exposure is associated with enhancing the permeability of intestinal epithelial cells in vitro [133], the prevalence of esophageal cancer [134], and hepatotoxicity [135,136]. DON, a major MT that potently inhibits the synthesis of proteins and nutrient intake [137], affects neuronal activity [138] and impairs male fertility [139]. The PMTDI has been set to 1 μg/kg BW for DON (and 3-ADON and 15-ADON). The potential health risks related to acute exposure to DON were evaluated by comparing the exposure percentiles with the acute reference dose (ARfD) of 8 μg/kg (3-ADON and 15-ADON) [140]. The provisional maximum tolerable daily intake (PMTDI) was set to 2 μg/kg BW for FUMs (FB1, FB2, FB3—alone or in combination) [141]. OTA, as a possible human carcinogen [130], induces genotoxic effects in HepG2 cells [142], impacts human renal cells [143], and causes acute kidney injuries [144]. A BMDL10 of 4.73 µg/kg BW/day for non-neoplastic effects and 14.5 µg/kg BW/day for neoplastic effects was set for OTA [145]. It was observed that ZEA potently causes an estrogenic syndrome in pigs and was identified as an endocrine disruptor in humans [146]. The current TDI for ZEA of 0.25 μg/kg BW/day established by the EFSA Panel for Contaminants in the Food Chain in 2011 is based on estrogenicity in pigs [147]. Commission Regulation 1881/2006 establishes maximum levels for MT contamination in foods [132]. Indicative maximum levels of 100 ng/kg BW for the sum of T-2 and HT-2 toxins have been recently issued [148] while BEA, ENs, FUS, MON, and NIV belong to the group of emerging MTs and are not present in any specific legislation yet. Relevant in vivo toxicity data are needed to perform a human risk assessment [149]. Monitoring studies for MT presence in foods have to be performed repeatedly to extract solid information about human exposure to contaminants.

According to Table 3, the sum of AFs AFB1, AFB2, and ABG1 was above the maximum level in bread products proposed by WHO and JECFA [113]. For white bread, the sum of AFs was 10.1 μg/kg, 7.7 μg/kg for multigrain bread, and 8.3 μg/kg for wholewheat bread. Risk exposure data have proven that DON is within the safe limits introduced by WHO and JECFA (Table 4). However, FUM contamination in Nigeria is 16,800 times higher than the maximum PMTDIs that have been accepted by WHO and JECFA. One of the measured OTA level exposures through sorghum in Nigeria was also above the limit for non-neoplastic effects (OTA = 13.22 μg/kg BW/day); however, the value was very close to the concentration that is potent for inducing neoplastic effects (Table 4).

### 4.2. Toxic Metals

TM pollution has been observed worldwide, is present in the environment, and can be dangerous to human life. The proposed definition occurring in the reviewed literature on toxic “heavy metals” is as follows: “naturally occurring metals having an atomic number greater than 20 and an elemental density greater than 5 g/cm^3^” [150,151,152,153]. Aluminum (Al), arsenic (As), cadmium (Cd), cobalt (Co), copper (Cu), mercury (Hg), manganese (Mn), nickel (Ni), and lead (Pb) are some of the predominant TMs and metalloids spread in the environment. Cu, iron (Fe), magnesium (Mg), Mn, and zinc (Zn) have been detected in all types of grain [154]. There has been prepared a specific classification that distinguishes essential TMs from non-essential ones. For instance, Cu, chromium (Cr) (III), Fe, and Zn are TMs that have been described as crucial for the proper functioning of a living organism, while As, Cd, Hg, and Pb have been classified as non-essential for any metabolic functions, as was reviewed previously [155].

**Figure 5:**
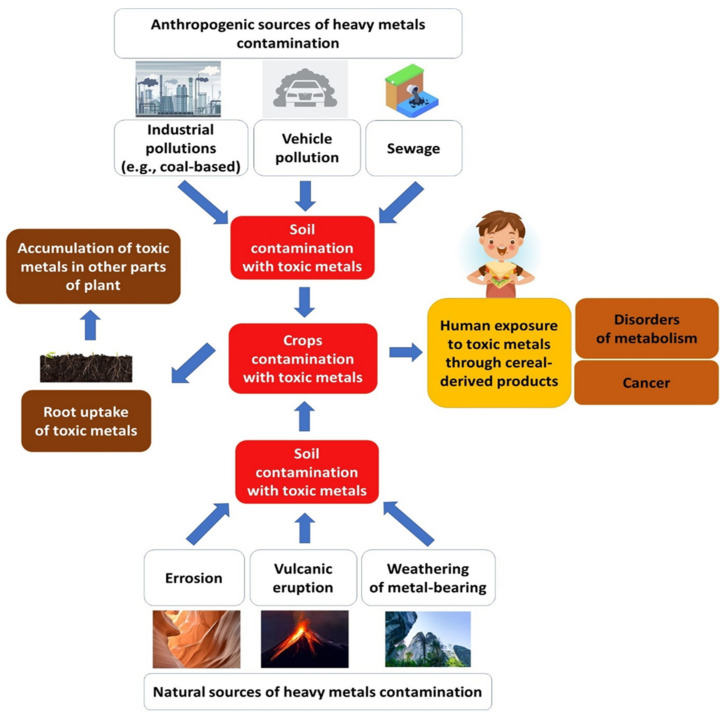
Natural and anthropogenic sources of heavy metal contamination in food crops and mechanisms of their uptake by plants, with the resulting adverse impacts on humans.

Regarding the TM sources, the natural sources of TMs include the weathering of metal-containing rocks and volcanic eruptions, while anthropogenic sources capture industrial emissions, mining, smelting, the exploitation of machines used in production and packaging steps, and agricultural activities such as the application of pesticides and phosphate fertilizers (Figure 5) [156]. Vehicle pollution is responsible for the release of TMs, e.g., Cd. The presence of TMs in the environment also ensures their presence in the food chain and exposes humans to TMs. Chronic exposure to TMs in the environment is highly not recommended to any living organisms [157]. Since TMs are part of the environment, soil acts as a source of TMs [158]. Moreover, TMs are absorbed by crop roots (TMs in the soil) and slowly accumulate in other parts of the plant (roots, leaves, and grains) causing negative effects on plant growth and, later, people’s health. However, TMs are also distributed in the air and water. Their presence and levels are mainly determined by environmental conditions (type of weather during cultivation, presence of rain, levels of soil contaminants). The scope of current TM inputs among various types of bread products is summarized in Table 5. Additionally, Table 6 presents data concerning the dietary intake of TMs in wheat and wheat-based products in various countries.

People are exposed to TMs through ingestion, inhalation, and dermal absorption. TMs, due to their tendency to bioaccumulate, are found to be very hard to metabolize in an organism. Various studies reported that TMs can negatively influence a great spectrum of human organs such as the lungs [164], bladder [165], heart [166,167], kidney, and liver [168], and can be responsible for exhibiting anti-androgenic activity [169] and induce haematological and histopathological changes [170]. Therefore, TMs have been classified in the top 20 list of dangerous substances by ATSDR [171]. The IARC classified TMs and metalloids in the following groups depending on their carcinogenicity to humans: Cd and Cd compounds along with As and inorganic As compounds into Group 1 (carcinogenic to humans); inorganic Pb into Group 2A (probably carcinogenic to humans); Pb, methylmercury, Ni, Co, and Co compounds into Group 2B (possibly carcinogenic to humans); Cr, Hg and inorganic Hg compounds, and organic Pb into Group 3 (not classified as carcinogenic; lack of human carcinogenicity exposure data) [172]. However, the WHO has announced that Cd, Pb, and Hg are in the top 10 chemicals that are the primary concern to public health [173]. Since then, TMs have been considered a public health concern worldwide. Some international organizations have advised performing dietary exposure studies and risk assessment calculations due to the lack of data on TMs in foodstuffs, which are crucial in public nutrition [174,175,176]. The EFSA revised the up-to-date health-based guidance value of each trace element. The BMDL10 value for Pb was set up to 0.63 µg/kg BW/day. The TWI of Cd was estimated to be 2.5 µg/kg BW/day, while for Hg, TWI was calculated to be 4.0 µg/kg BW/day [177,178,179].

Based on Table 6, the dietary intake of Pb, Cd, and Hg does not induce any harmful health effects on humans. The average dietary exposures are below the safe values set up by the EFSA. Since Cd and its compounds are classified as one of the most harmful TMs to humans, they have also not shown any vital biological function in any living organism or tissue [180,181]. Cereal-based food is the primary source of Cd, as more than 80% of vegetables and cereal are contaminated with Cd. Cd directly influences the oxidative stress of plants, leading to ROS production, inhibiting root growth, and inducing lipid peroxidation in roots [182].

### 4.3. Pesticides

**Figure 6:**
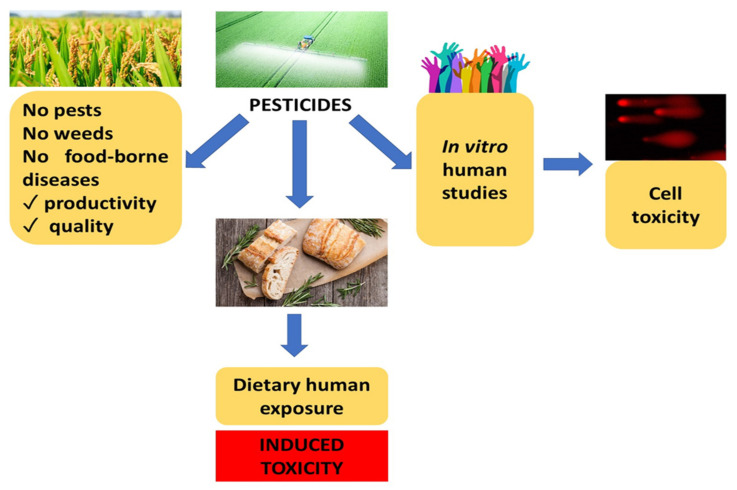
Positive and negative sides of pesticide use in agriculture.

The last, but no less important, source of bread contaminants that will be discussed in this review are pesticides. Nowadays, pesticides are applied to most crops in agriculture. It has been estimated that the usage of pesticides reaches about 4 million tons each year [183,184]. Pesticides include over 1000 different chemicals, which can be further classified depending on their targets, e.g., fungicides, herbicides, and insecticides. Organochlorine (e.g., dichloro-diphenyl-trichloroethane and benzene hexachloride) pesticides have been applied in agriculture since a long time ago, and now, their application has been eliminated in many countries due to their durability in nature. This elimination led to a more frequent usage of organophosphate pesticides (OPPs) and carbamates pesticides with a lower durability [185,186]. The most often applied OPPs cover dichlorvos, dimethoate, disulfoton, dursban (chlorpyrifos), fenthion, glyphosate, guthion (azinphos-methyl), malathion, methyl parathion, mocap (ethoprophos), parathion, phorate, pirimiphosmethyl, temephos, and tokuthion (prothiofosfenitrothion), while carbamates include bendiocarb, carbaryl (sevin), carbofuran +, methiocarb, and propoxur [186]. There is also another class of pesticides called pyrethroids including bifenthrin, cyfluthrin, deltamethrin, lambda-cyhalothrin, and permethrin, as reviewed by Nicolopoulou-Stamati et al. [186]. Pesticides oppose pests, weeds, or diseases, increase food production efficiency, and improve crop quality. On the other hand, pesticides also protect humans from food-borne diseases such as malaria, dengue fever, and schistosomiasis (Figure 6) [187].

However, OPPs and carbamates are still hazardous chemicals, and permanent human exposure to pesticides leads to adverse health effects [183,188]. Pesticide traces are present in various foodstuffs that are included in a daily diet and animal feed [188,189]. Human exposure to pesticides occurs via dermal absorption, inhalation, and oral ingestion [186,187]. Scientists’ hypotheses revealed that carbamates are potent carcinogens and mutagens. OPPs and carbamates inhibit acetylcholinesterase, resulting in the accumulation of acetylcholine at the endings of nerves. The mentioned accumulation is responsible for neurobehavioral dysfunction in the target pests [186,190]. It has been reported that pesticide elimination techniques such as washing and peeling will not reduce pesticide levels in foods [191]. However, the pesticide concentrations mostly do not reach the legislatively set-up MRLs [192]. MRLs are the highest concentrations of pesticides allowed to be present in foodstuffs [193]. Nonetheless, simultaneous human exposure to more than one pesticide in food is potent to induce synergistic effects, even though the specific pesticide concentrations alone are safe, as reviewed in Kortenkamp [194]. Epidemiological reports revealed that there have been specific associations between pesticides and some toxic effects. It has been reported that pesticides induce hepatotoxicity [195] and neurotoxicity [196], disrupt the endocrine system [197], induce alterations to erythrocytes and lymphocytes [198], enhance breast cancer [199], and disturb the homocysteine metabolism in pregnant women [200]. Palaniswamy et al.’s [201] study indicated that non-occupational overall pesticide exposure, length of exposure, and specific pesticides were associated with multiple biological markers of health in Finnish young adults. Those results need to be replicated to find the mechanism of pesticides’ course of action. It could be used in preclinical alterations or for adverse metabolic health effects triggered by pesticides. The metabolism of OPPs in human organisms starts with their conversion to dialkylphosphates (DAP) metabolites e.g., dimethylphosphate (DMP), dimethylthiophosphate (DMTP), dimethyldithiophosphate (DMDTP), diethylphosphate (DEP), diethylthiophosphate (DETP), and diethyldithiophosphate (DEDTP), and then they are excreted in the urine within a few days [202]. According to IARC [203], the herbicide glyphosate and the insecticides malathion and diazinon were classified as probably carcinogenic to humans (Group 2A). The insecticides tetrachlorvinphos and parathion were classified as possibly carcinogenic to humans (Group 2B). Generally, epidemiological exposure studies are very limited. However, dietary exposure to OPPs can be partially linked to their levels in urine. MRLs have been established to avoid a health risk for consumers from pesticide residues in food. The MRLs (mg/kg) of pesticides applied in cereals were set up in Annex I to Regulation (EC) No 396/2005 covered by Council Directive 86/362/EEC, and are as follows: carbaryl (sevin) (0.5); carbofuran + (0.02); dichlorvos (0.01); dimethoate (0.3); disulfoton (0.1); dursban (chlorpyrifos) (0.05); glyphosate (10); guthion (azinphos-methyl) (0.05); malathion (8); methyl parathion (0.02); parathion (0.05); and propoxur (0.05) [204,205]. The scope of the newest pesticides’ ubiquity in wheat-based products is summarized in Table 7. Additionally, Table 8 presents data concerning human exposure to pesticides in grains depending on the country. Generally, regarding the collected data in Table 7, all the pesticide concentrations in wheat-based products were in the safe range. However, one pesticide was found closer to the MRL than other pesticides. That pesticide is guthion, and its concentration has remained still 10 times lower than allowed MRLs.

## 5. Discussion and Conclusions

The toxicity studies of food processing contaminants in bread focus mainly on AA and furan derivatives, which are attractive substances in bread aroma but also highly toxic. According to collected data, most wheat-based bread is in the safe range of 50 µg of AA/kg. However, there is a specific correlation between wheat and non-wheat bread. Namely, according to several studies, non-wheat bread (wholemeal and rye, etc.) has elevated levels of AA in comparison to wheat ones [23,39,156]. Başaran et al. [156] research has shown that HMF (furan derivative) levels in tested food matrices have had skyrocketed values, much higher than the BMDLs for neoplastic effects induced by furan, while Ramírez-Jiménez et al. [212] assessed HMF levels in bakery products ranging from 9.5 to 151.2 mg/kg, exceeding even higher values than those observed by Başaran et al. [156] (87 mg/kg). However, in the study by Gülcan et al. [213], the HMF concentration in the bread sample was equal to 17 mg/kg. A short but significant list of research has been performed about PAHs which occur in baked bread. Ciecierska and Obiedziński [214] assessed the level of some PAHs in white bread, and values of 0.42 µg/kg and 0.09 µg/kg were measured for pyrene and 5-methylchrysene, respectively, while others [215] detected different PAHs, B[a]A and B[a]P, in cereals at the similar concentrations of 0–3.2 µg/kg and 0–0.11 µg/kg, respectively. The amounts of 3-MCPDs and their esters were in the safe range below the TDI (0.8 µg/kg BW/day) established by EFSA. In accordance with the results, it might be assumed that a 70 kg person can consume 56 µg of 3-MCPDs and 3-MCPDEs per day without any adverse health effects. In the study by Chung et al. [216], the 3-MCPD esters were measured at a level of 10.5 µg/kg in white bread. The amount of 2-MCPDs and their esters in wheat-based foodstuffs has not been established by any international organization yet. However, glycidol concentrations were in the safe range introduced by the EFSA Panel on Contaminants [104]. Chung et al. [216] have not detected glycidyl esters in white bread, while EFSA [104] has identified them in bread and bread rolls in values ranging from 0 to 510 µg/kg. However, it was not possible to find a single article about the contribution of pyrroles and pyridines to bakery goods. It seems that those groups of compounds are present in different heat-processed foodstuffs at a significantly higher level than in bread, and that is probably the reason why researchers are not interested in measuring their levels in bread-related products.

Soil is the central source of food crops and thereby can be subjected to MTs, TMs, and pesticides. From the overview of research data, it can be concluded that the environmental contamination of grains and bread products depends on several factors, including: (1) type of contaminant; (2) degree of food processing (raw samples, meaning wheat/cereal/grain, vs. already processed foods such as bakery goods); and (3) type of grains (white vs wholemeal), and (4) a country’s agriculture advancement.

MTs are a huge group of food contaminants. The information and performed research regarding MT contamination in grain-derived products are very extensive. In the case of AFs, white bread is more potent for contamination by higher concentrations of AFs than other types of bread [113,117]. However, in the case of bread contaminated with ENs and ZEA, the opposite phenomenon is found, as higher MT content has occurred in non-white bread [113,117]. Another aspect that directly influences MT concentration is food processing. Wheat (raw material before processing) has lower levels of MTs than processed ready-to-eat bakery products, but only in AF-contaminated products, while DON, T-2, HT-2, and NIV contamination is significantly higher in wheat than in processed bakery goods [3,113,117,119]. Corresponding to the gathered data, AFs levels in cereal products were above the level set up by Commission Recommendation 1881/2006. FUMs and OTA concentrations were above the accepted limits in Nigeria, while in developed countries, these levels were in the safe range [121]. A similar phenomenon was observed for DON concentration in rice. The DON level was also out of range in Iran [120]. These findings can indicate a real health threat to humans. In the study by Vaclavikova et al. [217], the ENs and DON levels were measured in two different flours, and the results revealed that ENs and DON contamination in flours ranged from 8 to 86 µg/kg and 13–96 µg/kg, respectively, while in a separate experiment, the mean DON level in wheat flour was 52 µg/kg, reaching a maximum of 622 µg/kg [218]. Zhao et al.’s [219] research showed the co-occurrence of multiple MTs in wheat grains, especially DON, together with 3-ADON and 15-ADON, and FUMs with other MTs produced by *Fusarium* spp. As a result, it has been recommended that grain food products should have been considered for regulation due to high concentrations. However, there is a lack of data concerning MTs’ fate in soils or an explanation of environmental exposure to MTs in general.

Elevated values for TM contamination were observed for wheat-based bread (Cd, Co, Cr, Mn, Pb), while for other types of bread, especially rye bread, the levels of Al and Hg were higher. In general, higher concentrations of TMs were found in non-wheat bread [156]. The TM (Cd, Hg, and Pb) concentrations in foods for which guidance levels were set up were below BMDL10 and TWI. In Başaran’s [156] study, the level of Cd was similar to Zioła-Frankowska et al. [157] at 3.3–49.5 µg/kg and 12.7–53.8, respectively. However, surprisingly low Cd concentrations were found by Ashot et al. [161]. The Cd level was 5.8 µg/kg. Those findings do not indicate any threat to human health. However, there is a lack of data regarding multi-metal toxicity in crops. The multi-metal transfer from soil to plants needs a specific approach that will determine the actual and/or total TM toxicity. Moreover, the epidemiological data remain for continued study. There are scanty data about the hazardous effects of TMs, and the level of TMs in the soil is permanently increasing. Pesticide usage in agriculture has many positive effects such as the elimination of food-borne diseases, but also negative ones. However, all the concentrations of pesticides reviewed in this paper are in the safe MRLs and did not pose any threat to human well-being. Despite that, pesticides were still present in wheat products.

To sum up, there is a long list of wheat and wheat-based product contaminants. Most of them are in safe ranges, but there are several contaminants that are above permissible levels and pose a real danger to human health (e.g., HMF and MTs-AFs, FUMs, OTA, DON). The question is what if upon consumption, humans are exposed to more than only one contaminant? What are the health risks then? Exposure to the large number of food contaminants such as MTs, TMs, pesticides, or Maillard reaction products consumed in one meal has not been assessed yet. To the authors’ best knowledge, cross-contaminant studies are still lacking. There is a huge gap in that research area. It might seem that humans are exposed to many food contaminants for every instance of consumption. The authors propose that the next step in toxicology research will be an assessment of the overall figure of contaminants in bread matrixes from different sources. Then, studies concerning mitigation strategies would be necessary. Nowadays, lactic acid bacteria (LAB) are used as a as probiotic microorganism to detoxify toxic substances. One of their applications is their addition to bread dough during bread making. It has been observed that the levels of toxic substances were significantly reduced after LAB treatment [220,221,222,223,224]. It would also be worth estimating if LAB can influence cross-contaminated food matrices, as would an evaluation of their ability to detoxify not only thermally induced toxicants but also contaminants from the environmental origin at the same time. The mitigation research is vital because the human body needs to metabolize those toxicants at the same time. Food contaminants will be metabolized and excreted from the body, but some of them can easily accumulate and cause adverse health effects. Cancer diseases are present in modern times and depend on DNA codes, but more importantly, they can be modulated by environmental factors including diet. There have been a great number of studies conducted concerning the relationship between human nutrition and cancer risk.

## Figures and Tables

**Table 1:** Food processing contaminants contribute to various bread-related products.

Food Processing Contaminant	Sample	Content [μg/kg]		Reference
		Mean	Min-Max	
AA	Multigrain bread	79	79	[22]
	White bread	87	87	
	Whole wheat bread	77	77	
	Wholemeal bread	84	84	
	Rye bread	83	83	
	Toasted wheat bread	22	10–34	[38]
	Wheat bread	21	<20–30	[26]
	Rye bread	31	<20–42	
	Cornbread	27	<20–34	
	Mixed bread	25	<20–39	
	Bread	57	31–90	[23]
	Bread rolls	52	42–67	
	Friselle	358	306–454	
	Wholemeal bread	61	44–88	
	Wholemeal friselle	384	328–450	
	Toast bread	134	45–246	[20]
	Non-wheat bread	43	4–163	[39]
	Wheat bread	27	6–65	
5-HMF	Multigrain bread	16,000	16,000	[22]
	Wholemeal bread	23,000	23,000	
	Whole wheat bread	10,000	10,000	
	Rye bread	17,000	17,000	
	White bread	37,000	37,000	
2-Pentylfuran	Bread crust	270	258–282	[40]
	Chinese sourdough steamed bread	902	180–1625	[41]
Furfural	Bread crust	216	183–249	[40]
Naphthalene	Chinese sourdough steamed bread	65	21–109	[41]
PAHs	Toasted bread	1.5	0–3	[42]
3-MCPD	White bread	5	5	[43]
	Bread	120	120	[44]
3-MCPDE	White bread	1	0–2	[43]
	Bread and bread rolls	29	23–26	[45]
2-MCPD	White bread	<10	<10	[43]
	Bread	30	30	[44]
2-MCPDE	White bread	1	1–2	[43]
	Bread and bread rolls	14	10–20	[45]
Glycidol	Bread	650	650	[44]
GE	White bread	3	3	[43]
	Bread and bread rolls	8	8	[45]

**Table 2:** Dietary exposure to food processing contaminants in bread products in various countries.

Food Processing Contaminant	Country	Sample	Average Dietary Exposure	Reference
AA	Turkey	Multigrain bread	0.22 μg/kg BW/day	[22]
	Spain	White bread	0.31 μg/day	[43]
	Chile	Bread	0.22 μg/kg BW/day	[46]
	Romania		14 μg/kg	[47]
	Croatia	Wheat bread	0.16 μg/kg BW/day	[26]
	Italy	Bread	100 μg/kg	[48]
	Slovenia	Toast bread	0.21 μg/kg BW/day	[20]
	Poland	Wheat bread	0.31 μg/kg BW/day	[39]
	Portugal	Bread	787 μg/kg	[49]
	Latvia	Wheat bread	0.89 μg/person/day	[50]
	China	Bread	35 μg/kg	[51]
	Finland	Rye bread	51 μg/kg	[52]
	Korea	Bread	33 μg/kg	[53]
HMF	Turkey	Multigrain bread	87,000 μg/kg BW/day	[22]
5-HMF	Iran	Flat bread	12,000 μg/kg BW/day	[54]
PAHs	Romania	Toasted bread	0.005 μg/kg BW/day	[42]
3-MCPD	Spain	White bread	0.30 μg/day	[43]
2-MCPD			0.31 μg/day	
3-MCPDEs			0.04 μg/day	
2-MCPDEs			0.06 μg/day	
GE			0.19 μg/day	

**Table 3:** Up-to-date research on mycotoxins’ presence in different wheat-based products.

Mycotoxin	Sample	Content [μg/kg]		Reference
		Mean	Min-Max	
AFB1	Wheat	1	1	[117]
	White bread	5.6	4.2–7.1	[113]
	Whole wheat bread	6.1	6.1	
	Multigrain, oatmeal, corn, kamut, rye, lactose and gluten-free	5.2	5.2	
AFB2	White bread	3.6	3.1–4.2	
	Whole wheat bread	2.2	0.5–3.2	
	Crustless white bread	4.1	1.0–5.3	
	Crustless whole wheat bread	1.8	0.8–3.5	
AFG1	White bread	2.9	2.9	
	Multigrain, oatmeal, corn, kamut, rye, lactose and gluten-free	2.5	2.5	
Enniatin A (ENA)	Wheat wholemeal grains	15.7	3.0–28.4	[118]
Enniatin A1 (ENA1)	Whole wheat bread	2.4	2.2–2.6	[113]
	Multigrain, oatmeal, corn, kamut, rye, lactose and gluten-free	2.6	2.6	
Enniatin B (ENB)	Wheat wholemeal grains	408.7	30.1–787.3	[118]
	White bread	9.8	2.0–18.7	[113]
	Whole wheat bread	16.5	1.3–41.1	
	Multigrain, oatmeal, corn, kamut, rye, lactose and gluten-free	16.9	0.4–54.0	
	Crustless white bread	14.8	1.4–8.7	
	Crustless whole wheat bread	10.6	1.0–31.0	
Enniatin B1 (ENB1)	Wheat wholemeal grains	130.3	4.8–255.8	[118]
	White bread	2.9	0.2–6.0	[113]
	Whole wheat bread	6.5	1.5–14.8	
	Multigrain, oatmeal, corn, kamut, rye, lactose and gluten-free	6.3	0.2–14.0	
	Crustless white bread	4.6	0.4–13.0	
	Crustless whole wheat bread	5.1	2.4–13.0	
DON	Bread	41.0	39.4–42.6	[3]
	Wheat	369	369	[117]
		44.3	1.1–955	[119]
15-ADON	Wheat wholemeal grains	33.6	10.9–55.8	[118]
	Bread	5.6	3.8–7.3	[3]
	Wheat	18.6	8.9–30	[119]
3-ADON	Bread	8.4	6.9–9.9	[3]
	Wheat	7.5	2.7–12	[119]
FUMs		117	117	[117]
OTA	Bread	2.7	2.5–2.9	[3]
	Wheat	3	3	[117]
T-2	Bread	4.6	3.3–5.4	[3]
	Wheat	25	25	[117]
HT-2	Bread	19.3	11.5–27.1	[3]
	Wheat	51.7	24.7–98.5	[119]
NIV	Bread	50.8	17.5–84.0	[3]
	Wheat	55.9	40.0–64.3	[119]
ZEA	Bread	11.2	9.6–12.9	[3]
	Wheat	34	34	[117]
		90.7	11.7–300	[119]
	White bread	56.8	36–80.0	[113]
	Whole wheat bread	48.8	29.0–100.0	
	Multigrain, oatmeal, corn, kamut, rye, lactose and gluten-free	178.6	27.0–905.0	
	Crustless white bread	96.8	40.0–214.0	
	Crustless whole wheat bread	67.0	30.0–135.0	

**Table 4:** Risk assessments of mycotoxin distribution in grain products depending on the country.

Mycotoxin	Country	Sample	Average Dietary Exposure	Reference
AFB1	Iran	Rice	10 ng/kg BW/day	[120]
AFT			16 ng/kg BW/day	
AFs	Nigeria	Sorghum	0.08 mg/kg BW/day	[121]
AFB1	Spain	White bread	1.06 ng/kg BW/day	[113]
AFB2				
AFG1				
DON	Iran	Rice	242.71 ng/kg BW/day	[120]
	Brazil	Wheat flour	0.05 μg/kg BW/day	[122]
	Portugal		0.24 µg/kg BW/day	[123]
	China	Whole wheat	0.65 μg/kg BW/day	[124]
3-ADON			0.02 μg/kg BW/day	
15-ADON			0.008 μg/kg BW/day	
ENA1	Spain	White bread	1.06 ng/kg BW/day	[113]
ENB				
ENB1				
FB1	Iran	Rice	118 ng/kg BW/day	[120]
FUMs	Nigeria	Sorghum	33.58 mg/kg BW/day	[121]
FB1	Brazil	Wheat flour	0.07 μg/kg BW/day	[122]
FB2				
FB3				
OTA	Iran	Rice	0.7 ng/kg BW/day	[120]
	Nigeria	Sorghum	13.22 μg/kg BW/day	[121]
	Brazil	Wheat flour	0.01 μg/kg BW/day	[122]
	China	Whole wheat flour	0.003 μg/kg BW/day	[124]
	Spain	White bread	2.60 ng/kg BW/day	[113]

**Table 5:** Current toxic metal inputs among various types of bread products.

Toxic Metal	Sample	Content [μg/kg]		Reference
		Mean	Min-Max	
Al	Multigrain bread	9670	7110–12,500	[156]
	Wholemeal bread	10,900	7660–16,800	
	Whole wheat bread	7730	6140–12,10	
	Rye bread	18,100	7210–123,000	
	White bread	7720	5600–13,500	
	Various types of bread samples	3620	2060–6560	[159]
	Homemade bread	296,500	249,000–344,000	[158]
As	Multigrain bread	9.7	4.8–31.6	[156]
	Wholemeal bread	15.3	11.4–25.7	
	Whole wheat bread	17.3	7.9–48.6	
	Rye bread	21.6	13–28.3	
	White bread	16.4	7.8–56.8	
	Various types of bread samples	5.1	2.9–16.2	[159]
	Homemade bread	67.5	54–81	[160]
Boron (B)	Various types of bread samples	2760	90–6850	[159]
Calcium (C)a		440,000	310,000–1,920,000	
Cd	Multigrain bread	15.9	13.4–49.5	[156]
	Wholemeal bread	17.0	11.4–20.6	
	Whole wheat bread	12.6	3.3–19.3	
	Rye bread	11.8	4–16.7	
	White bread	11.7	3. 9–17.8	
	Various types of bread samples	35.9	12.7–53.8	[159]
	White bread	5.8	5.8	[161]
Co	Multigrain bread	20.9	<0.06–69.1	[156]
	Wholemeal bread	21.6	7.7–30	
	Whole wheat bread	2.8	<0.06–47.6	
	Rye bread	6.5	<0.06–25	
	White bread	1.6	<0.06–22.3	
Cr	Multigrain bread	72.4	38.5–535	
	Wholemeal bread	65.2	45.1–126	
	Whole wheat bread	47.3	37.2–70.0	
	Rye bread	89.1	75.2–280	
	White bread	50	21.5–174	
	Various types of bread samples	62.9	36.8–266.1	[159]
	Homemade bread	425	370–510	[160]
Cu	Multigrain bread	4040	2220–6640	[156]
	Wholemeal bread	3900	3300–4210	
	Whole wheat bread	3530	2910–4920	
	Rye bread	3060	2710–3890	
	White bread	2890	2640–4590	
	Various types of bread samples	1660	930–3850	[159]
	White bread	0.002	0.002	[161]
Fe	Various types of bread samples	15,130	7420–39,200	[159]
Hg	Multigrain bread	0.29	<0.26–0.61	[156]
	Wholemeal bread	0.28	0.16–0.94	
	Whole wheat bread	<0.26	<0.26–0.38	
	Rye bread	0.08	<0.26–0.69	
	White bread	<0.26	<0.26–0.71	
	Various types of bread samples	2.63	0.93–8.63	[159]
	White bread	8.6	8.6	[161]
Potassium (K)	Various types of bread samples	3,310,000	1,840,000–4,750,000	[159]
Mg		50,000,000	240,000–1,420,000	
Mn	Multigrain bread	28,900	8850–42,000	[156]
	Wholemeal bread	25,900	20,700–32,800	
	Whole wheat bread	23,900	16,700–43,700	
	Rye bread	17,700	16,400–24,300	
	White bread	15,500	11,600–43,100	
	Various types of bread samples	7280	2800–15,830	[159]
Molybdeum (Mo)	White bread	0.03	0.03	[161]
Sodium (Na)	Various types of bread samples	6,780,000	5,940,000–8,770,000	[159]
Ni	Multigrain bread	597	89.8–2720	[156]
	Wholemeal bread	281	249–427	
	Whole wheat bread	269	203–498	
	Rye bread	339	198–699	
	White bread	228	141–642	
	Various types of bread samples	120	10–410	[159]
	White bread	0.02	0.02	[161]
	Homemade bread	1415	1330–1500	[160]
Pb	Multigrain bread	22.6	4.6–83.0	[156]
	Wholemeal bread	11.2	1. 5–68.4	
	Whole wheat bread	11.2	<0.1–149	
	Rye bread	46.2	20–121	
	White bread	27.9	<0.14–97.6	
	Various types of bread samples	38.5	29.5–98.6	[159]
	White bread	0.1	0.1	[161]
	Homemade bread	160	140–180	[160]
Selenium (Se)	Various types of bread samples	15.1	4.8–53.1	[159]
Zn		8890	5940–15,120	

**Table 6:** Dietary intake of toxic metals in wheat and wheat-based products in various countries.

Toxic Metal	Country	Sample	Average Dietary Exposure	Reference
Al	Turkey	Multigrain bread	25.8 μg/kg BW/day	[156]
	Poland	Various types of bread samples	9.84 μg/kg BW/day	[159]
As	Turkey	Multigrain bread	0.06 μg/kg BW/day	[156]
	Poland	Various types of bread samples	0.148 μg/kg BW/day	[159]
Cd	Turkey	Multigrain bread	0.03 μg/kg BW/day	[156]
	Pakistan	Wastewater irrigated wheat	1.04 μg/kg/day	[162]
	Poland	Various types of bread samples	0.081 μg/kg BW/day	[159]
	China	Wheat grain	0.45 μg/kg BW/day	[163]
Co	Turkey	Multigrain bread	0.3 μg/kg BW/day	[156]
Cr	Turkey		0.19 μg/kg BW/day	
	Pakistan	Wastewater irrigated wheat	1.17 μg/kg/day	[162]
	Poland	Various types of bread samples	0.399 μg/kg BW/day	[159]
Cu	Turkey	Multigrain bread	8.39 μg/kg BW/day	[156]
	China	Wheat grain	11.52 μg/kg BW/day	[163]
Hg	Turkey	Multigrain bread	<0.01 μg/kg BW/day	[156]
	Poland	Various types of bread samples	0.013 μg/kg BW/day	[159]
Mn	Turkey	Multigrain bread	53.4 μg/kg BW/day	[156]
Ni	Turkey		0.74 μg/kg BW/day	
	Pakistan	Wastewater irrigated wheat	0.96 μg/kg/day	[162]
	Poland	Various types of bread samples	0.615 μg/kg BW/day	[159]
Pb	Turkey	Multigrain bread	0.09 μg/kg BW/day	[156]
	Poland	Various types of bread samples	0.024 μg/kg BW/day	[159]
	China	Wheat grain	0.13 μg/kg BW/day	[163]
Zn			60.45 μg/kg BW/day	

**Table 7:** Newest pesticides’ ubiquity in wheat-based products.

Pesticide	Sample	Content		Reference
		Mean	Min-Max	
3-hydroxylcarbofuran	Wheat	<0.30 ng/g	<0.30 ng/g	[186]
Bifenthrin	Whole wheat flour	7.9 μg/kg	<1.0–76.1 μg/kg	[206]
Carbaryl (sevin)	Wheat	<0.30 ng/g	<0.30 ng/g	[186]
	Agricultural products	0.0189 mg/kg	0.0064–0.0471 mg/kg	[207]
Carbendazim	Whole wheat flour	212.8 μg/kg	<1.0–4279.7 μg/kg	[206]
Carbofuran	Wheat	<0.25 ng/g	<0.25 ng/g	[186]
	Agricultural products	0.0966 mg/kg	0.0017–0.3562 mg/kg	[207]
Cyhalothrin	Whole wheat flour	5.8 μg/kg	<3.0–48.3 μg/kg	[206]
Dichlorvos		<0.1 μg/kg	<0.1–3.1 μg/kg	
	Wheat	<0.26 ng/g	<0.26 ng/g	[186]
	Bread	1.2 µg/kg	1.1–1.3 µg/kg	[208]
Disulfoton	Wheat	<0.20 µg/kg	<0.20 µg/kg	
Dursban (chlorpyrifos)	Agricultural products	0.0016 mg/kg	0.0016 mg/kg	[207]
	Wheat	<0.50 µg/kg	<0.50 µg/kg	[208]
Guthion (azinphos-methyl)		4.0 µg/kg	4.0 µg/kg	
Lambda cyhalothrin	Bread	0.15 µg/kg	0.0–0.3 µg/kg	
Malathion	Agricultural products	0.0285 mg/kg	0.0027–0.0739 mg/kg	[207]
Methiocarb	Wheat	<0.30 ng/g	<0.30 ng/g	[188]
	Agricultural products	0.0068 mg/kg	0.0064–0.007 mg/kg	[207]
Methyl parathion	Wheat	<0.20 ng/g	<0.20 ng/g	[186]
Mocap (ethoprophos)		<0.16 ng/g	<0.16 ng/g	
	Agricultural products	0.0051 mg/kg	0.0009–0.0127 mg/kg	[207]
Nicosulfuron	Whole wheat flour	1.9 μg/kg	<1.0–19.0 μg/kg	[206]
Parathion		<5.0 μg/kg	<5.0 μg/kg	
Propiconazole		1.1 μg/kg	<1.0–16.8 μg/kg	
Propoxur	Wheat	<0.24 ng/g	<0.24 ng/g	[186]
	Agricultural products	0.022 mg/kg	0.0056–0.0523 mg/kg	[207]
Tebuconazole	Whole wheat flour	4.4 μg/kg	<1.0–45.4 μg/kg	[206]
	Agricultural products	0.0314 mg/kg	0.0032–0.0979 mg/kg	[207]
Tokuthion (prothiofos)	Wheat	<0.15 ng/g	<0.15 ng/g	[186]

**Table 8:** Human exposure to pesticides in grains depending on the country.

Pesticide	Country /Continent	Sample	Average Dietary Exposure	Reference
Carbaryl (sevin)	Nigeria	Cereal	2.05 ng/kg/day	[186]
	Korea	Agricultural products	1114.81 µg/person/day	[209]
Carbofuran	Brazil	Fruits, vegetables, grains and cereals	0.10 µg/kg	[210]
	Canada		0.08 µg/kg	
	Czech Republic		0.08 µg/kg	
	Italy		0.01 µg/kg	
	USA		0.08 µg/kg	
	Serbia	Fruit juice	76.7 mg/kg BW	[211]
	Nigeria	Cereal	3.41 ng/kg/day	[186]
	Korea	Agricultural products	185.9 µg/person/day	[209]
Dichlorvos	Australia	Fruits, vegetables, grains and cereals	0.01 µg/kg	[210]
	Brazil		0.2 µg/kg	
	Canada		0.2 µg/kg	
	Czech Republic		0.08 µg/kg	
	Italy		0.05 µg/kg	
	USA		0.5 µg/kg	
	Nigeria	Cereal	4.75 ng/kg/day	[186]
	Korea	Agricultural products	91.37 µg/person/day	[209]
Disulfoton	Nigeria	Cereal	1.37 ng/kg/day	[186]
	Korea	Agricultural products	223.53 µg/person/day	[209]
Dursban (chlorpyrifos)	Nigeria	Cereal	5.97 ng/kg/day	[186]
	Korea	Agricultural products	263.77 µg/person/day	[209]
Malathion			338.1 µg/person/day	
Methiocarb	Nigeria	Cereal	2.05 ng/kg/day	[186]
	Korea	Agricultural products	56.63 µg/person/day	[209]
Methyl parathion	Nigeria	Cereal	1.37 ng/kg/day	[190]
Mocap (ethoprophos)			1.09 ng/kg/day	
Tokuthion (prothiofos)			4.18 ng/kg/day	
Guthion (azinphos-methyl)			14.34 ng/kg/day	
Phorate	Australia	Fruits, vegetables, grains and cereals	0.15 µg/kg	[210]
	Brazil		0.33 µg/kg	
	Czech Republic		0.14 µg/kg	
	Italy		0.07 µg/kg	
	USA		0.21 µg/kg	
Propoxur	Nigeria	Cereal	1.64 ng/kg/day	[186]

## Data Availability

Not applicable.
